# Effects of chlorogenic acid on antimicrobial, antivirulence, and anti-quorum sensing of carbapenem-resistant *Klebsiella pneumoniae*

**DOI:** 10.3389/fmicb.2022.997310

**Published:** 2022-12-13

**Authors:** Lingbo Wang, Yi Zhang, Yan Liu, Mengxin Xu, Zhuocheng Yao, Xiaodong Zhang, Yao Sun, Tieli Zhou, Mo Shen

**Affiliations:** ^1^Department of Clinical Laboratory, The First Affiliated Hospital of Wenzhou Medical University and Key Laboratory of Clinical Laboratory Diagnosis and Translational Research of Zhejiang Province, Wenzhou, Zhejiang, China; ^2^Department of Microbiology, Zhejiang Provincial Center for Disease Control and Prevention, Hangzhou, China; ^3^Department of Medical Lab Science, School of Laboratory Medicine and Life Science, Wenzhou Medical University, Wenzhou, Zhejiang, China

**Keywords:** chlorogenic acid, carbapenem-resistant, hypervirulent, *Klebsiella pneumoniae*, quorum sensing

## Abstract

The rise in infections caused by the hypervirulent carbapenem-resistant *Klebsiella pneumoniae* (hv-CRKP) is an emergent threat to public health. We assessed the effects of chlorogenic acid (CA), a natural phenolic compound, on antibacterial, antivirulence, and anti-quorum sensing (QS) of hv-CRKP. Five hv-CRKP were selected for antimicrobial susceptibility test and confirmed to carry virulence genes and carbapenem-resistant genes by polymerase chain reaction (PCR). Subsequently, a series of time-kill assay, determinations of protease activity and capsule content, biofilm-related experiment, scanning electron microscopy (SEM) and transmission electron microscope (TEM) observation, *G. mellonella* infection model, quantitative real-time PCR (qRT-PCR) of QS-related genes and biofilm formation genes, as well as AI-2 binding test were conduct to verify the effect of CA on hv-CRKP. Five CRKP strains showed varying degrees of resistance to antibacterial agents. All strains carried the *bla*_*KPC*–2_ gene, primarily carrying *rmpA2*, *iucA*, and *peg-344*. CA showed no effect on CRKP growth at the 1/2 minimum inhibitory concentration (MIC), 1/4 MIC, and 1/8 MIC, CA could reduce the production of extracellular protease and capsular polysaccharides, and improve the survival rate of larvae in *Galleria mellonella* (*G. mellonella*) infection model. By means of crystal violet staining and scanning electron microscopy experiments, we observed that CA can inhibit the formation of CRKP biofilm. On the quantitative real-time PCR analysis, the expression of the *luxS*, *mrkA* and *wbbm* genes in most CRKP strains appeared downregulated because of the CA treatment. Besides, CA significantly inhibited the effect of AI-2 activity of BB170. Our study suggests that CA can be an effective antimicrobial, antivirulent compound that can target QS in hv-CRKP infections, thus providing a new therapeutic direction for treating bacterial infections.

## 1 Introduction

*Klebsiella pneumoniae* (*K. pneumoniae*), an important opportunistic Gram-negative bacteria and one of the most common pathogenic bacteria in hospitals (Ballen et al., 2021), is responsible for the frequent incidence of a serious hospital- and community-acquired infections, including urinary tract infections, lung infections, and bloodstream infections (M Campos et al., 2020; Hu et al., 2021). Studies have shown that the clinical detection rate of *K. pneumoniae* is second only to *Escherichia coli*, exhibiting a year-on-year increase (Yang F. et al., 2019). In recent years, the resistance of *K. pneumoniae* to antibacterial drugs has been increasing, therefore, carbapenems, because of their broad antibacterial spectrum and strong antibacterial activity, are being increasingly used to treat serious bacterial infections (Doi, 2019). However, the wide use and abuse of carbapenems in clinical practice is giving rise to carbapenem-resistant *K. pneumoniae* (CRKP), even causing nosocomial infection outbreaks, posing considerable challenges to clinical treatment (Candan and Aksoz, 2015; Xu et al., 2017; Tang et al., 2020; Zhao et al., 2020).

According to virulence characteristics, *K. pneumoniae* can be classified as classic *K. pneumoniae* (cKp) and hypervirulent *K. pneumoniae* (hvKp) (Bialek-Davenet et al., 2014). The hvKp strain carries plasmids pLVPK-encoding virulence genes such as *rmpA* and *rmpA2*, and the highly transmissible hvKp infections often occur in multiple (Russo and Marr, 2019), responsible for a variety of life-threatening infections, such as liver abscess, endomyelitis, and meningitis (Cubero et al., 2016; Wang et al., 2020). To date, because of selective pressure of antibiotics, multidrug resistance isolates and hvKp have been detected in clinical practice, in particular, the emergence of hypervirulent CRKP (hv-CRKP), which is highly challenging to anti-infection treatment protocols (Tang et al., 2020). Therefore, new treatment strategies to effectively inhibit hv-CRKP is emergently required.

Quorum sensing (QS) is a mechanism of communication between bacteria that depends on the bacterial population density; QS aids regulation of bacterial biological functions, such as, secretion of pathogenic extracellular protease and toxins, biofilm formation, and resistance development to various antibacterial drugs (Pena et al., 2019; Wu et al., 2020). *K. pneumoniae* primarily use LuxS/AI-2 as the self-induction factor of its QS system, and the AI-2 QS system of *K. pneumoniae* has a regulatory effect on biofilm formation and lipopolysaccharide synthesis (De Araujo et al., 2010; Papenfort and Bassler, 2016). Thus, inhibiting QS will potentially help in fighting the pathogenic bacteria.

Quorum sensing inhibitors (QSIs) by interfering with the QS system can reduce virulence factors and pathogenic effects of the bacteria without affecting its growth of bacteria, thus effectively inhibiting infections caused by the pathogen (Kalia et al., 2019). In addition, QSIs can act as synergists in delaying antimicrobial resistance development. The combination of QSIs and antibacterial drugs can restore bacterial sensitivity to previously tolerated drugs, thus helping reduce the effective dose and improve the efficiency of antibacterial drugs (Ham et al., 2021). Natural QSIs mainly exist in bacteria, fungi, animals, and plants, and marine organisms (Kalia, 2013). Chlorogenic acid (CA), a polyphenolic compound richly found in fruits and vegetables, has antitumor, anti-inflammatory, and antiviral activities (Santana-Galvez et al., 2017). CA can inhibit the biofilm formation, virulence factor, and QS system of *Pseudomonas aeruginosa* (Xu et al., 2022). However, relatively few studies on the antibacterial and antivirulence sensing of CRKP. The emergence of hv-CRKP has created an urgency to develop a new strategy to deal with related infections.

Therefore, we aimed to explore the effects of CA on hv-CRKP and its potential antivirulence mechanism.

## 2 Materials and methods

### 2.1 Bacterial strains and antimicrobial susceptibility testing

A total of 5 strains were selected from 20 CRKP strains isolated from the Wenzhou Medical University from January to May 2020. These isolates were identified by matrix-assisted laser desorption/ionization-time of flight mass spectrometry (MALDI-TOF MS; bioMérieux, Lyons, France). In addition, duplicate strains isolated from the same part of the same patient were removed during strains collection. The minimum inhibitory concentrations (MICs) of meropenem, imipenem, ertapenem, ampicillin, ceftriaxone, ceftazidime, cefepime, ciprofloxacin, levofloxacin, gentamycin, tobramycin, colistin, and CA were determined by the microdilution broth method with cation-adjusted Mueller–Hinton broth as per the guidelines of the Clinical and Laboratory Standards Institute (CLSI, 2021). In addition, CA (purity ≥ 98%, Beijing Soleibao Co., Ltd., Beijing, China) was dissolved in dimethyl sulphoxide (DMSO). ATCC 27853 and ATCC 25922 were used for quality control.

### 2.2 Detection of virulence and resistance genes of CRKP

The DNA of CRKP isolates were extracted using the BioFlux Bacterial DNA Extraction Kit (Bioflux, Tokyo, Japan). The virulence genes (*rmpA*, *rmpA2*, *iucA*, *iroB*, and *peg-344*) and carbapenem-resistant genes (*bla*_KPC–2_, *bla*_NDM_, *bla*_VIM_, and *bla*_OXA–232_) were amplified by the polymerase chain reaction (PCR) with specific primers (Supplementary Table 1). The amplified PCR products were sequenced by Shanghai Majorbio Bio-Pharm Technology Co. (Shanghai, China). Further sequence alignment was performed to analyze any possible gene mutations by BLAST on the National Center for Biotechnology Information (NCBI).^1^

### 2.3 Time-kill assay

The time-kill assay was performed to evaluate the effect of CA on the growth of CRKP, as described previously with some minor modifications (Lu et al., 2013). Briefly, the five CRKP strains were inoculated into 20 mL of cation-adjusted Mueller-Hinton broth (CAMHB) containing different concentrations of CA (1/2, 1/4, and 1/8 MIC). Tubes containing the LB medium alone served as the negative control. The bacterial suspensions were incubated at 37°C with moderate shaking for 2, 4, 6, 12, and 24 h. According to the growth rate of bacteria, appropriate dilution concentration was prepared. At the corresponding time points, 100 μL of the bacterial suspension was spotted on the Mueller-Hinton (MH) agar plates, and the colony forming unit (CFU) was counted after incubating the plate overnight at 37°C. All studies were conducted in duplicate.

### 2.4 Protease activity assay

We measured the diameter of the precipitation ring on Luria-Bertani (LB) skim-milk agar plate at 37°C for 18–36 h to test the protease activity semi-quantitatively. The diameter of the precipitation ring reflected the protease activity (Phrommao et al., 2011).

We also quantitatively measured the protease activity of bacterial supernatants treated with different concentrations of CA (1/2 and 1/4 MIC) by a modified azocasein assay (Nicodeme et al., 2005). Briefly, 1 mL of culture supernatant was combined with 1 ml of 10 mM Tris–Cl buffer (pH 7.5), 3 mg/mL of azocasein, and 0.5 mM CaCl_2_ for the protease test. With mild shaking, the reaction mixture was incubated at 37°C for 1 h. The reaction was terminated with the addition of 0.4 mL of 10% trichloroacetic acid. Then, the mixture was centrifuged at 10,000 × *g* for 10 min, and the absorbance of the supernatant was measured at 420 nm. By dividing the A420 values by the A600 (cell density) of the various bacterial cultures, the specific proteolytic activity unit (U) was computed.

### 2.5 Quantification of capsule assay

Capsules were quantified as described previously, albeit with some modifications (Yang X. et al., 2019). Briefly, 500 μL of the cultured bacterial suspensions were resuspended and adjusted to 10^6^ CFU/mL, and 1.2 mL sodium tetraborate in sulfuric acid was added to the resuspensions placed on an ice bath and incubated for 5 min at 100°C, and then left on the ice for 10 min. A 20-μL volume of 1.5 mg/mL *m*-hydroxydiphenyl was then added to its mixture and mixed well. After a 5-min incubation at room temperature, the absorbance at 590 nm was measured. The glucuronic acid content was determined with reference to a standard curve of glucuronic acid and expressed as μg/10^8^ CFU. The results were presented as the mean of data of three independent experiments.

### 2.6 Transmission electron microscopy (TEM)

In order to better understand the changes in the capsule of CRKP when treated with different concentrations of CA, a transmission electron microscopy (stocktickerTEM; Hitachi HT7700, Germany) examination was performed, using the experimental method as previously described with some modifications (Wang et al., 2022). In short, fresh bacterial cells of *K. pneumoniae* FK 8123 with different treatments of CA (none, 1/2 MIC, and 1/4 MIC) were tested with stocktickerTEM. 10 μL of bacterial solution was applied to copper mesh and allowed to connect for around 10 min after fresh bacterial cells had been rinsed with phosphate-buffered saline (PBS) once. Filter paper was used to absorb any extra bacterial fluid before samples were dyed for 1 min with 1% uranyl acetate dihydrate applied to copper mesh.

### 2.7 Evaluation of the antivirulence of CA in *G. mellonella* infection model *in vivo*

We detected the efficacy of CA in *Galleria mellonella* infection *in vivo* through a survival assay, as described previously with some modifications (Brackman et al., 2011). FK 8036 was selected for the experiment. Healthy larvae weighing at least 250 mg and free of any gray markings were selected at random for the experiments. Then, 10 *G. mellonellas* were randomly selected from each group. Overnight cultures of CRKP strain were washed with PBS and further adjusted with PBS to a concentration of 1 × 10^4^ CFU/mL. The insects infected with PBS were used as control. We considered an average haemolymph volume of 50 μL and the increase in the volume contributed to bacterial and antibiotic injections (each 10 μL). For example, 10 μL of an antibiotic solution with 7-time greater concentration was injected into the larvae. Then, 10 μL of the bacterial solution was injected into the rear left proleg of *G. mellonella* by using a microinjector, followed by the test of the CA (1/2 and 1/4 MIC) of 7-time within 2 h of infection. Then *G. mellonellas* at 37°C and recorded their survival rate after 24, 48, 72, 96, 120, 144, and 168 h. All experiments were conducted in triplicate. Death was considered when the larvae repeatedly failed to respond to a physical stimuli. Kaplan–Meier analysis and log-rank test were performed to analyze the survival rate of *G. mellonella* larvae.

### 2.8 Biofilm-formation inhibition assay

Biofilm-formation assays were performed in 96-well polystyrene microtiter plates, as previously described (Yang X. et al., 2019) with some modifications. A single colony on the blood plate was shaken overnight in 3 mL of fresh LB broth medium at 37°C. Then, the culture was adjusted to 0.5 McFarland with sterile normal saline and further diluted by 1:100 in LB broth and dispensed in a 96-well microtiter plate with 1/2, 1/4, and 1/8 MIC CA. The 96-well plates were incubated at 37°C for 24 h. Then, the cell suspension was removed and the plates were washed twice with 1 × PBS (Sigma-Aldrich, Milan, Italy) and inverted to dry at the room temperature. Next, 200 μL of 1% crystal violet (CV) solution (Beijing Solarbio Biotechnology Co., Ltd., China) was added to the wells for 15 min. After staining, CV was removed and the wells were washed thrice with 1 × PBS. After the plate dried naturally at room temperature, the bound CV was solubilized by adding 200 μL of ethanol–acetone (95:5 v/v) solution. The absorbance of CV was read at 595 nm on a microplate reader (Multiskan FC). All tests were performed in triplicate, with at least three independent experiments.

### 2.9 Mature biofilm eradication assay

We further detected the eradication effect of CA on mature biofilms, as suggested elsewhere (Yu et al., 2020), albeit with some modifications. Briefly, the 5 CRKP overnight cultures in 3 mL of fresh LB broth medium were adjusted to 0.5 McFarland with sterile normal saline, followed by further dilution to 1:100 in LB broth medium and dispensing in a 96-well microtiter plate. After 24 h of static incubation at 37°C (the formation of mature biofilms), the supernatant was discarded and the plates were washed thrice with 0.9% saline to remove the unattached cells. Next, fresh LB broth containing 2, 4, and 8 MIC CA was, respectively, added to each well (200 μL/well). The LB broth medium without antimicrobials was served as control, and the 96-well plates were incubated for 24 h at 37°C. Subsequent staining and treatment were performed as described previously. Each assay was performed in triplicate.

### 2.10 Scanning electron microscopy (SEM)

To evaluate the morphological changes of the bacterial biofilm after different treatments (Hemmati et al., 2020), FK 8036 was randomly selected for SEM. The overnight culture in LB medium incubated at 37°C was adjusted to 0.5 McFarland with sterile normal saline, and sterile coverslips were placed in each well of a 6-well plate to which 100 μL of diluted culture plus 1,900 μL CA (1/2 and 1/4 MIC) were added. Blank LB broth served as the negative control group. Biofilms were grown on coverslips at 37°C for 18–24 h. After which, the bacterial culture was removed and washed with 1 × PBS. The biofilm samples were fixed with 2.5% glutaraldehyde fixation solution in a fresh 6-well plate and incubated at 4°C for 4 h, after which the samples were subjected to a gradient series of ethanol (30, 50, 70, 90, and 100% v/v) for 10 min. All samples were then dried for 2 h and observed by SEM (S-3000N, Japan).

### 2.11 Quantitative real-time PCR (qRT-PCR)

The quantification of the expression of *rmpA2*, *iucA*, *luxS*, *mrkA*, *wzm*, *wbbm*, and *treC* in 5 CRKP was performed by quantitative real-time PCR (qRT-PCR), while *16SrRNA* served as the standardized reference gene for CRKP. Briefly, the strain was cultured in the LB broth for 16–18 h, to which CA (1/2 MIC) was added and the mixture was incubated for 6 h. The total RNA from the cultured strain was extracted by using a commercial RNA extraction kit (Tiangen Biotech, Beijing, China). The gene expression was quantified by the 2^–ΔΔCt^ method. The experiments for each gene were performed in triplicate. The primer sequences were listed in the Supplementary Table 2.

### 2.12 AI-2 binding assays *in vitro*

The steps for detecting the activity of culture supernatant can refer to reference and be modified as appropriate (Panayi et al., 2022). The steps are as follows: *Vibrio harveyi* (*V. harveyi*) BB170 is inoculated in autoinducer bioassay (AB) culture medium and cultured overnight at 30°C. Before detection, the overnight culture solution of *V. harveyi* BB170 was diluted with fresh AB medium at a ratio of 1:5,000, and shaken well. A total of 900 μL diluted *V. harveyi* BB170 cell were mixed with 100 μL bacterial supernatant treated with different concentrations of CA (1/2, 1/4, and 1/8 MIC). At the same time, *V. harveyi* and the supernatant of AB culture medium were used as positive and negative controls. Continue shaking culture at 30°C, then take 200 μL/well to the black 96-well flat base plate every 1 h for 5 h, and use the multifunctional microplate reader (BioTek) to detect its bioluminescence intensity. When the luminous intensity of the negative control reaches the minimum, the luminous value of the positive control is set to 100%. The data result is expressed by relative activity.

### 2.13 Statistical analysis

The statistical significance was evaluated by using one-way analysis of variance (ANOVA) with *post hoc* analysis using Tukey’s test to evaluate significant differences between groups. Student’s *t*-test and log-rank test were used to determine the statistical significance of gene expression and survival rate, respectively. For all analyses: *P < 0.05, **P < 0.01, ***P < 0.001, and ns *P* > 0.05.

## 3 Results

### 3.1 Microbiological characteristics of CRKP isolates

We identified the biochemical characteristics of 5 CRKP strains (FK 7887, FK 7917, FK 8002, FK 8036, and FK 81230) and found that their oxidase, indole, methyl red, hydrogen sulfide (H_2_S) production, and motility test were negative, while Voges–Proskauer and Simmons citric acid test were positive, which accorded with the biochemical characteristics of *K. pneumoniae*. Five CRKP strains in our study did not have hypermucoviscous phenotype.

### 3.2 Antimicrobial susceptibility testing

We used CA and common clinical antibiotics to determine the MICs of five CRKP strains. For all the five CRKP strains, the MIC of CA was 10,240 μg/mL (Table 1); for all five CRKP strains exhibited different degrees of resistance to clinical antibiotics.

**TABLE 1:** Minimum inhibitory concentration (MIC) value of carbapenem-resistant *Klebsiella pneumoniae* against clinical antibiotics and chlorogenic acid (CA).

Strains	MIC (μg/mL)													
	
	MEM	IPM	ETP	ATM	AMP	CRO	CAZ	FEP	CIP	LEV	GEN	TOB	COL	CA
FK 7887	16	32	>256	>256	>256	64	>128	>128	>32	>64	>256	128	128	10,240
FK 7917	256	128	>256	>256	>256	>128	>128	>128	>32	>64	1	1	0.5	10,240
FK 8002	128	32	256	>256	>256	>128	>128	>128	>32	>64	>256	>256	0.5	10,240
FK 8036	>128	>16	>8	>64	>32	>64	>64	>64	>4	>8	>16	>16	0.5	10,240
FK 8123	>128	1	1	0.5	>256	2	>128	64	1	0.5	1	1	0.5	10,240

MEM, meropenem; IPM, imipenem; ETP, ertapenem; ATM, aztreonam; AMP, ampicillin; CRO, ceftriaxone; CAZ, ceftazidime; FEP, cefepime; CIP, ciprofloxacin; LEV, levofloxacin; GEN, gentamicin; TOB, tobramycin; COL, colistin; CA, chlorogenic acid.

### 3.3 Virulence and drug resistance genes

**FIGURE 1:**
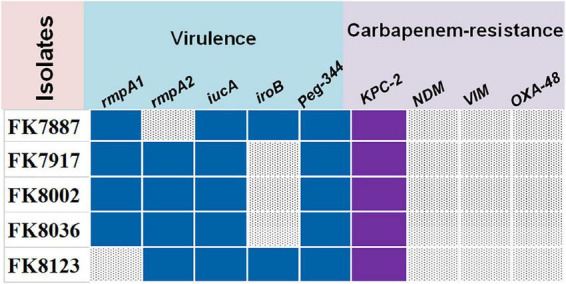
The virulence and carbapenem-resistant genes of five carbapenem-resistant *Klebsiella pneumoniae* (CRKP) strains. Blue color represents virulence genes, purple color represents carbapenem-resistant genes, and gray color represents don’t carry the corresponding gene.

The strains that carried their respective virulence genes are as follows: FK 7887 carried *rmpA*, *iucA*, *iroB*, and *peg-344*; FK 7917, FK 8002, and FK 8036 strains carried *rmpA*, *rmpA2*, *iucA*, and *peg-344*; and FK 8123 strain carried virulence genes *rmpA2*, *iucA*, *iroB*, and *Peg-344*; and all five CRKP strains only carried carbapenem-resistant gene *bla*_KPC–2_ (Figure 1).

### 3.4 Time-kill analysis

**FIGURE 2:**
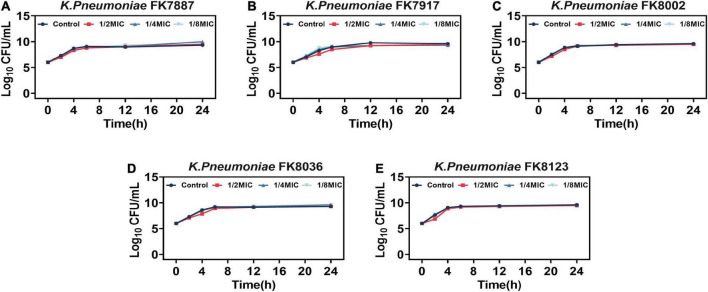
**(A-E)** Effects of different concentrations of chlorogenic acid (CA) on the growth of five carbapenem-resistant *Klebsiella pneumoniae* (CRKP) strains. Different color represents different concentrations of CA. The dark blue represents the control group, the red represents 1/2 MIC, the light blue represents 1/4 MIC, and the green represents 1/8 MIC.

1/2, 1/4, and 1/8 MIC CA did not affect bacterial growth (Figure 2), suggesting that CA has no effect on the growth of CRKP at sub-MICs (*P* > 0.05).

### 3.5 Inhibition of protease production

**FIGURE 3:**
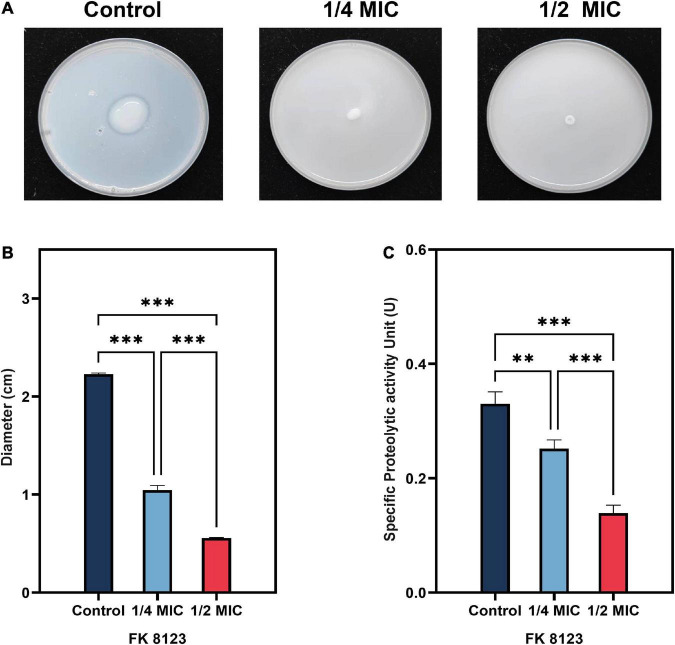
Protease inhibition assay. **(A)** Milk agar containing a series of concentrations of chlorogenic acid (CA) [1/2 and 1/4 minimum inhibitory concentration (MIC)] supplemented with carbapenem-resistant *Klebsiella pneumoniae* (CRKP); **(B)** Average diameter of five CRKP isolates; **(C)** Specific protease activity unit. The “**” means *P* < 0.01, “***” means *P* < 0.001.

*Klebsiella pneumoniae*–secreted protease has the ability to destroy host tissue proteins, and is an important virulence factor for the pathogenicity of this organism. The protease secretion detection of the five CRKP strain showed that only the FK 8123 strain produced protease. Therefore, the FK 8123 strain was used to assess the effect of CA on the strain’s protease production capacity, results are shown in Figure 3. When CA was not used, the diameter of the colony growth was larger and the hydrolysis ring was obvious, indicating the ability of the FK 8123 strain to produce protease. After the addition of 1/2 and 1/4 MIC CA, the hydrolysis ring of the strain became significantly smaller (*P* < 0.05), indicating that CA had a certain inhibitory effect on protease production. Besides, we assessed the ability of the FK 8123 supernatant to degrade casein with control group, 1/2 and 1/4 MIC CA treatment. With an increase in CA concentration under test conditions, the ability of the FK 8123 supernatant to degrade casein decreased (*P* < 0.05), further indicating that CA had a certain inhibitory effect on protease production.

### 3.6 Inhibition of capsular polysaccharide

**FIGURE 4:**
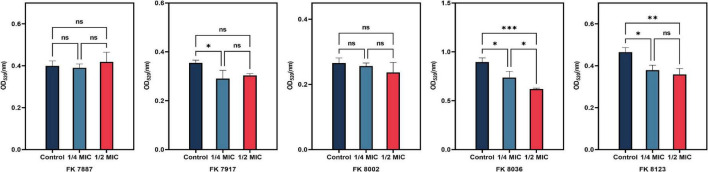
Capsular polysaccharide inhibition assay. Different color represents different concentrations of chlorogenic acid (CA) [1/2 and 1/4 minimum inhibitory concentration (MIC)] supplemented with carbapenem-resistant *Klebsiella pneumoniae* (CRKP). “*” means *P* < 0.05, “**” means *P* < 0.01, “***” means *P* < 0.001, and “ns” means *P* > 0.05.

**FIGURE 5:**
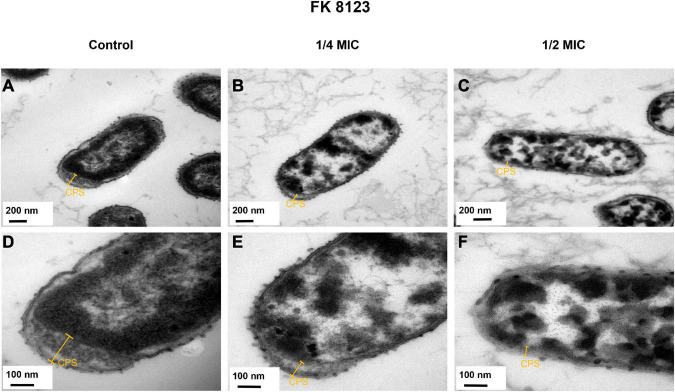
Transmission electron microscopy (TEM) images of the effects of chlorogenic acid (CA) treatment on the capsular of FK 8123. **(A)** LB broth control group, 30,000×; **(B)** 1/4 minimum inhibitory concentration (MIC) CA monotherapy, 30,000×; **(C)** 1/2 MIC CA monotherapy, 30,000×; **(D)** LB broth control group, 80,000×; **(E)** 1/4 MIC CA monotherapy, 80,000×; **(F)** 1/2 MIC CA monotherapy, 80,000×.

The presence of capsular polysaccharide in *K. pneumoniae* contributes to its high-viscosity and high-virulence phenotype. We assessed the effect of CA on the capsular polysaccharide production of the five CRKP strains. CA had a significant inhibitory effect on the capsular polysaccharide production of the FK 7917, FK 8036, and FK 8123 strains (*P* < 0.05) (Figure 4). We conducted the TEM experiment to better comprehend the changes in the capsule of CRKP treated with CA. Under the treatment of CA (1/2 or 1/4 MIC), the capsule became thinner, further demonstrating the inhibitory effect of CA on the capsular (Figure 5).

### 3.7 Virulence assays *in vivo* by *G. mellonella* larvae infection model

**FIGURE 6:**
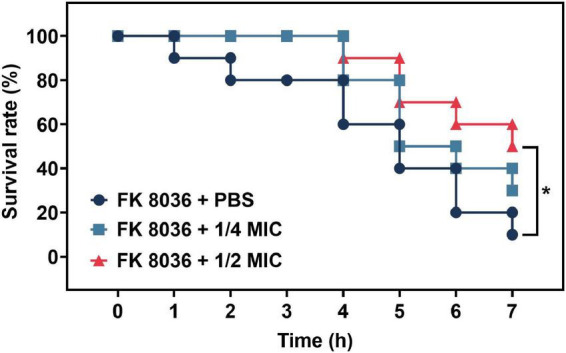
Survival rate of *Galleria mellonella* after 7 days of treatment with different concentrations of chlorogenic acid (CA) [1/2 and 1/4 minimum inhibitory concentration (MIC)] against FK 8036. Kaplan–Meier analysis and log-rank test were performed to analyze the survival rate of *G. mellonella* larvae.

The number of surviving *G. mellonella* larvae in the FK 8036 + PBS control group gradually decreased within 7 days after infection with *K. pneumoniae* (Figure 6). Compared with the survival rate of the control group, the survival rate of *G. mellonella* larvae treated with 1/2 MIC CA was significantly improved (*P* < 0.05).

### 3.8 Inhibition of biofilm formation

**FIGURE 7:**
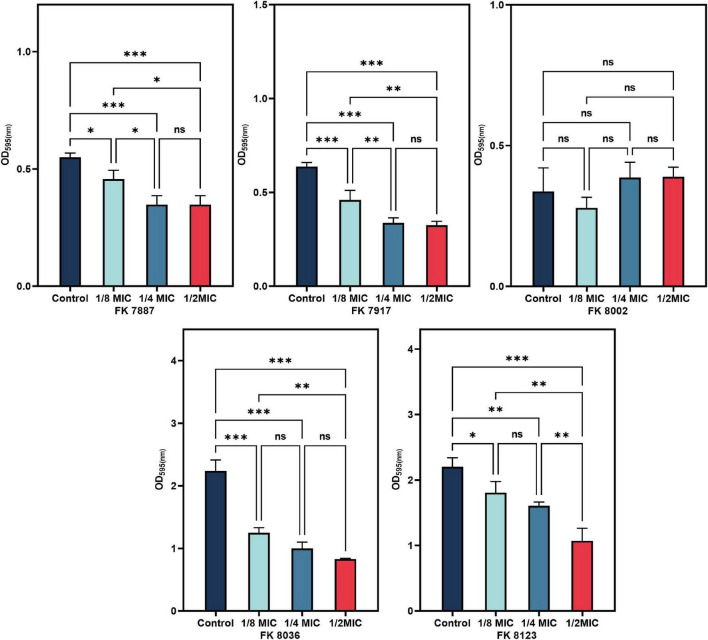
The inhibitory effect of biofilm formation of five carbapenem-resistant *Klebsiella pneumoniae* (CRKP) treated with chlorogenic acid (CA) at 1/2, 1/4, and 1/8 minimum inhibitory concentration (MIC). “*” Means *P* < 0.05, “**” means *P* < 0.01, “***” means *P* < 0.001, and “ns” means *P* > 0.05.

All the five CRKP strains had the weakest biofilm formation ability in the presence of 1/2 MIC CA (Figure 7). The inhibitory ability of CRKP strains was significantly different (*P* < 0.05), except that of the FK 8002 strain. This insignificant effect on the FK 8002 strain after treatment could be because of this strain’s weak biofilm forming ability.

### 3.9 Mature biofilm eradication assays

**FIGURE 8:**
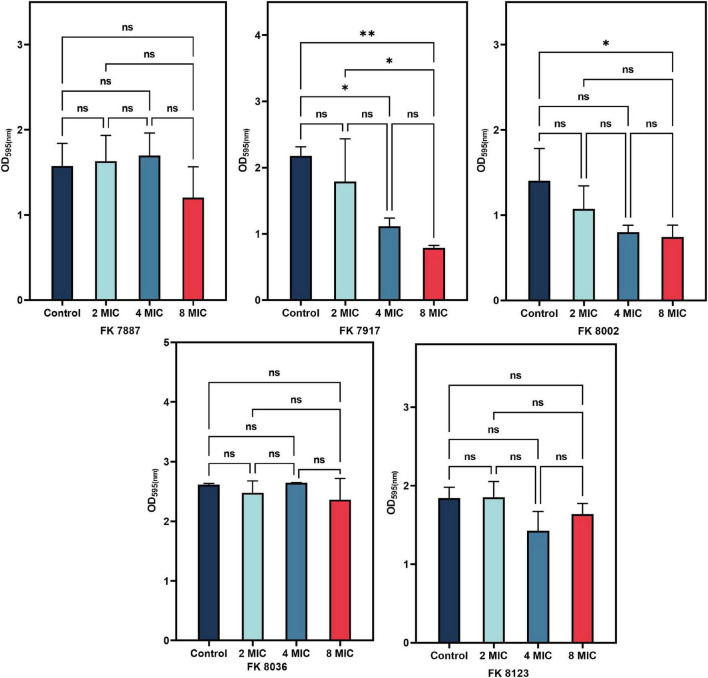
Radiation effect of chlorogenic acid (CA) on five carbapenem-resistant *Klebsiella pneumoniae* (CRKP) mature biofilm at 1/2, 1/4, and 1/8 minimum inhibitory concentration (MIC). “*” Means *P* < 0.05, “**” means *P* < 0.01, and “ns” means *P* > 0.05.

A bacterial biofilm is difficult to eradicate because of the persistent presence of bacteria. Therefore, to assessed the ability of CA to remove the biofilm formed by *K. pneumoniae*, we selected different concentrations of CA for experiments; and the results were shown in Figure 8. The ability of CA to scavenge the biofilms formed by *K. pneumoniae* was not significant, except that of FK 7917 at 4 and 8 MIC and that of FK 8002 at 8 MIC (*P* < 0.05). The ability of each CA concentration measured to remove biofilms of other strains was not significant.

### 3.10 Biofilm morphology

**FIGURE 9:**
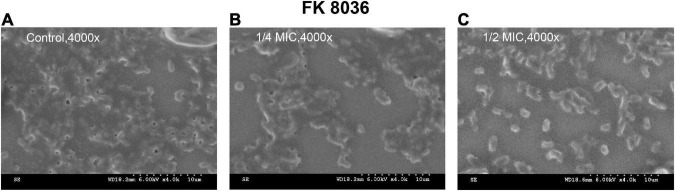
Scanning electron microscopy (SEM) images of the effects of chlorogenic acid (CA) treatment on the bacterial number and biofilm formation of FK 8036. **(A)** LB broth control group, 4,000×; **(B)** 1/4 minimum inhibitory concentration (MIC) CA monotherapy, 4,000×; **(C)** 1/2 MIC CA monotherapy, 4,000×.

We used SEM to assess the biofilm morphology and distribution of the FK 8036 strain after CA treatment; the results were shown in Figure 9. The form and quantity of the FK 8036 strain-formed biofilm were different before and after CA treatment. In the strain treated with the LB broth, the number of colonies, and their network structure were large and the bacterial morphology appeared complete. Whereas, in the strains treated with different concentrations of CA, the formed biofilm was weak, the network structure of colony growth appeared damaged, the strains were scattered, and the bacteria were aggregated in small parts.

### 3.11 CA regulated the expression of QS-related genes and biofilm formation genes

**FIGURE 10:**
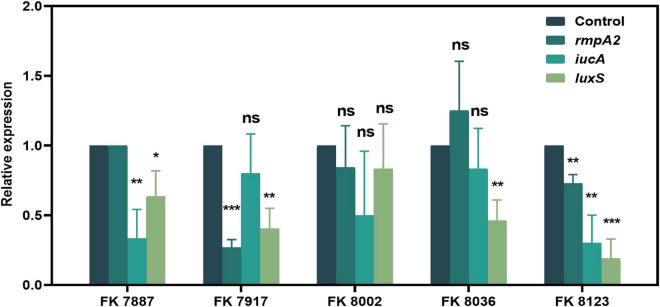
The expression of quorum sensing (QS)-related genes of five carbapenem-resistant *Klebsiella pneumoniae* (CRKP) strains in different treatment groups. The strain FK 7887 did not carry the *rmpA2* gene, hence this result will not be discussed. “*” Means *P* < 0.05, “**” means *P* < 0.01, “***” means *P* < 0.001, and “ns” means *P* > 0.05.

**FIGURE 11:**
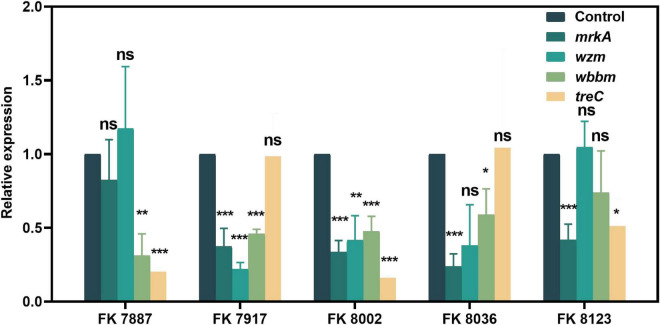
The expression of biofilm formation genes of five carbapenem-resistant *Klebsiella pneumoniae* (CRKP) strains deal with different treatment groups. “*” Means *P* < 0.05, “**” means *P* < 0.01, “***” means *P* < 0.001, and “ns” means *P* > 0.05.

We used qRT-PCR to further verify the inhibitory of CA on the virulence genes, QS-related genes and biofilm formation genes of CRKP isolated from clinics. The effects of CA on gene expression helped elucidate its regulatory effect on strain virulence, biofilm formation, and QS at the transcriptional level. As shown in Figures 10, 11, CA had different inhibitory effects on different genes in the five CRKP strains, with 1/2 MIC CA treatment, the expression levels of the *luxS* genes and biofilm formation regulators *mrkA* and *wbbm* were downregulated in five CRKP strains (*P* < 0.05).

### 3.12 Inhibition of AI-2 activity

**FIGURE 12:**
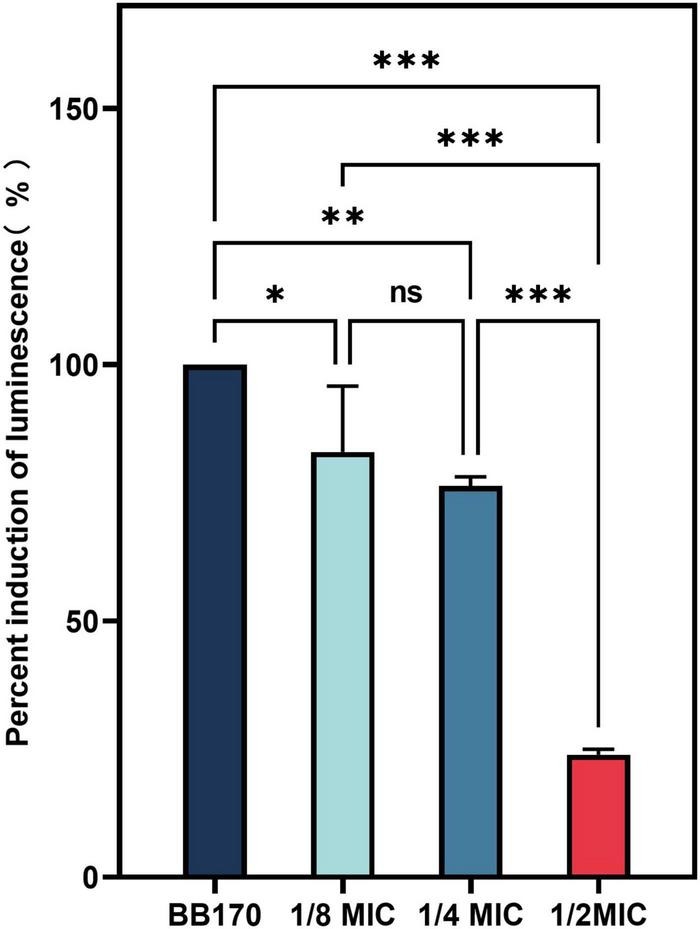
The effect of chlorogenic acid (CA) on the percent induction of luminescence of *Vibrio harveyi*. “*” Means *P* < 0.05, “**” means *P* < 0.01, “***” means *P* < 0.001, and “ns” means *P* > 0.05.

We used *V. harveyi* BB170 to investigated the inhibition of CA on AI-2 activity. As shown in Figure 12, CA significantly inhibited the effect of AI-2 activity of BB170 (*P* < 0.05).

## 4 Discussion

*Klebsiella pneumoniae*, an important Gram-negative opportunistic pathogen, widely exists in environment and on mucosal surfaces of animals and humans; it is responsible for urinary tract infection, bacteremia, lung infection, and liver abscess (Bengoechea and Sa Pessoa, 2019). According to its virulence, *K. pneumoniae* can be classified as cKp and hvKp, with hvKp being stronger than cKp in virulence and pathogenicity, hvKp infection occurs usually appears in multiple parts of the body and is highly transferable and transmission, therefore, intervention and control strategies are urgently required (Shon et al., 2013). In general, highly virulent isolates have high adhesion, and protease and capsular polysaccharide levels (Russo and Marr, 2019). The QS system is a communication system between microbial cells, and it regulates the virulence, ability for biofilm formation, and other pathogenic factors of bacterial strains. The QS allows the pathogenic bacteria to achieve high cell density before the virulence determinant can be expressed, helping it carry out a coordinated attack against host defense (Kai, 2018). Therefore, the inhibition of virulence factors and virulence-related QS is vital to control the pathogenicity of bacterial strains.

Because of the antibiotic selective pressure caused by its long-term use, clinical drug-resistant *K. pneumoniae* strains have emerged, with CRKP appearing more frequently and poses considerable challenges to clinical treatment. Many scholars attempted to develop and use non-traditional antibacterial drugs, such as antimicrobial peptides, bacteriophages, and plant extracts that had certain antibacterial and virulence and biofilm inhibition effects, in place of traditional antibacterial drugs (Khan et al., 2021; Witherell et al., 2021). The QSIs can decrease the strain virulence factors, biofilm formation ability of the strain; therefore, they proved to be ideal for treatment purposes (Ham et al., 2021). Substances such as quercetin, resveratrol, and 6-gingerol (Quecan et al., 2019; Ham et al., 2021). However, some QSI retain some form of toxicity, hindering their optimal clinical application. Therefore, further investigation to find suitable QSIs is required.

Chlorogenic acid, also known as 5-Caffeoylquinic acid, is found in many plants and hence part of the human diet (Singh et al., 2022). A natural phenolic compound, it is frequently used in food- and conventional medicine–related fields because of its low toxicity level. CA has been approved by China Food and Drug Administration (CFDA) for phase I and phase II clinical trials (Nwafor et al., 2022). In our previous studies (Xu et al., 2022), CA had a good inhibitory effect on the motility and virulence phenotypes of multidrug resistant *P. aeruginosa*. The growth and virulence phenotypes of *K. pneumoniae*, especially CRKP, needed to be further studied.

The five CRKP strains exhibited different antimicrobial resistance to different antibacterial drugs, better sensitivity to gentamicin, tobramycin, and colistin and weak sensitivity to non-antibacterial CA whose MIC value was > 10,240 μg/mL. Based on this study, the detection results of *peg-344*, *iroB*, *iucA*, *rmpA*, and *rmpA2* virulence genes of the five CRKP strains, most strains carry virulence genes of varying degrees, suggesting that the incidence of *K. pneumoniae* with high virulence and multidrug resistance characteristics is increasing in clinical practice. Efforts need to be made toward timely detection of infections caused by the multidrug-resistant *K. pneumoniae*, because of its high pathogenicity mediated by its high virulence phenotype. In contrast, detection results of carbapenem-resistant genes *bla*_KPC–2_, *bla*_NDM_, *bla*_VIM_, and *bla*_OXA–48_ showed that all the five strains carried *bla*_KPC–2_, a result consistent with that of previous reports. The prevalence of carbapenem-resistant genes expressed by *K. pneumoniae* in different countries and regions is diverse and not similar, but in *K. pneumoniae* isolated from China, the *bla*_KPC–2_ strain remains the most important prevalent epidemic type.

Because the growth of the strain influences the virulence phenotype of the strain, we used different CA concentrations to assess its effect on the growth of the five CRKP strains to test its effect, on the virulence phenotype of the strains. We found that CA at concentrations of 1/2, 1/4, and 1/8 MIC had no effect on the growth of CRKP. The extracellular products of *K. pneumoniae*, including various extracellular enzymes play a vital role in the infection of the bacterial strain in the host (Mayville et al., 1999). In addition, the high capsule production of *K. pneumoniae* can convert the colony into a high-viscosity phenotype that has a protective effect on phagocytes and human defensin-mediated bactericidal activity (Hoh et al., 2019). Our results indicated that CA reduced the generation of extracellular protease of the FK 8123 strain, the only strain producing protease among the five CRKP strains. We will include more strains in future studies to verify their ability for protease inhibition of CA. Moreover, CA had a significant inhibitory effect on the capsular polysaccharide production of the FK 7917, FK 8036, and FK 8123 strains, but its effect on that of the FK 7887 and FK 8002 strains was not obvious, which we speculated could be related to the capsular polysaccharide content secreted by the strain or to the characteristics of the strain itself. TEM showed that under the treatment of CA (1/2 or 1/4 MIC), the capsule became thinner. *G. mellonella* infection model was used to evaluate the effect of many non-antibacterial drugs on the virulence of bacteria, therefore, we used the CRKP infection model of *G. mellonella* to test the effect of CA on the virulence of the strain. Our results showed that 1/2 MIC CA could inhibit the virulence of the strain to a certain extent and improve the survival rate of *G. mellonella*. In our previous studies, we have shown that 1/4 MIC (2,560 μg/mL) CA had no cytotoxicity on RAW 264.7 macrophages (Xu et al., 2022), and the pre-experiment conducted in the *G. mellonella* infection assay showed that the CA used in this study was not toxic to the survival rate of *G. mellonella*, compared with the control group (data not shown), indicating CA has no cytotoxicity and could be further developed for clinical treatment use. Because of the complex mechanism of *in vivo* infection model, the inhibitory effect of CA on an *in vivo* strain virulence model needs to be further verified using other animal models and infection models.

The increase in pathogenic invasiveness and drug resistance to infection caused by *K. pneumoniae* has a significant correlation with biofilm. A biofilm, is a complex microbial community that can be formed on surfaces and can form dynamic structures (Karatan and Watnick, 2009). Many persistent, recurrent and refractory chronic infectious diseases, such as endocarditis, urinary tract infection, and chronic otitis media, are closely related to the formation of biofilms (Davey and O’Toole, 2000). *K. pneumoniae* has the ability to form biofilms on the inner surface of catheters and other medical devices, making these conducive to the development of invasive infection (Singhai et al., 2012). The biofilm formation process of *K. pneumoniae* is closely related to the factors mediating virulence, such as pili, and capsular polysaccharide and lipopolysaccharide, which are essential for maintaining the stability of biofilm structure and for inter-cell communication. The QS system coordinates and controls the signal and response of gene expression in bacterial population, thus playing an important role in bacterial adhesion and regulation of the formation of bacterial biofilm. However, deletion of QS genes may not reduce biofilm formation or virulence, therefore, the research on the interference with the signal molecules of the QS system this has certain significance.

Many plant extracts, such as resveratrol and quercetin, have good anti-toxicity, anti-biofilm, and anti-QS activities (Vasavi et al., 2017). The results of our study showed that CA can inhibit the biofilm formation ability of most the CRKP except the FK 8002 strain. This could be attributed to the fact that the biofilm formation ability of FK 8002 itself is weak; therefore, the inhibition effect on its ability is not significant under different concentrations of CA. SEM images showed that the network structure of the biofilm was destroyed after CA treatment and the number of colonies reduced and appeared dispersed, suggesting that CA does have a certain inhibitory effect on the process of biofilm formation, this result can help the potential use of CA in treatment of medical device–related diseases by resisting biofilm formation. In addition, our results showed that CA (8, 4, and 2 MIC) had poor clearance effect on the formed biofilm, which may be related to the existence of resistant persistent bacteria in the biofilm. Due to the biofilm were affected by many factors, it is worth further to explore the mechanism behind their inhibitory effect.

In the QS system, bacteria communicate with each other by secreting chemical signaling molecules. Through QS, many bacteria regulate the expression of various physiological functions, such as strain locomotion, virulence, biofilm formation, and bacteriocin production. The QS system of *K. pneumoniae* mainly uses type-2 QS, namely, AI-2, which is typically involved in the expression regulation of virulence-related factors, secretion system, regulatory proteins, and peptides, as the media molecule for communication with other bacterial population, and it is primarily regulated by the *luxS* gene (Zhu and Mekalanos, 2003). In addition, the virulence genes of *K. pneumoniae* are also correlated with their virulence phenotypes. *rmpA2* can regulate the synthesis of polysaccharide in the outer capsular membrane to make the strain have high stickiness; the siderophore-related gene *iucA* is also closely related to the high stickiness of *K. pneumoniae*. Our results showed that CA downregulated the expression levels of *luxS* genes in most CRKP, but showed weak effect on the expression levels of *rmpA2* and *iucA*, suggesting that CA may affect the virulence phenotype or biofilm formation of *K. pneumoniae* by downregulating the *luxS* gene. However, although CA downregulated the expression level of the *luxS* in the FK 8002 strain, there was no significant difference, which may be the reason why CA could not effectively inhibit the biofilm formation of the FK 8002. Furthermore, Balestrino et al. (2005) revealed that the *luxS* mutant could form a mature biofilm but one that had reduced capacity to develop microcolonies, mostly in the early steps of the biofilm formation, indicting the deletion of QS genes may not influence biofilm formation. Pan et al. (2021) found that no major differences were observed between the wild-type and the *luxS* mutant in regard to outer membrane protein profiles, biofilm formation, exopoly saccharides (EPS) production, or intracellular survival, which indicating that the deletion of QS genes may not reduce biofilm formation. The genes of *mrkA*, *wzm*, *wbbm*, and *treC* are all involved in the biofilm formation of *K. pneumoniae* (Vuotto et al., 2017), *wzm* and *wbbM* are lipopolysaccharide (LPS) synthesis-related genes and participate in LPS production. *MrkA* controls the production of type 3 fimbriae (Gual-de-Torrella et al., 2022), and *treC* participates in the production of capsular polysaccharide (Devanga Ragupathi et al., 2020). We further investigated the expression of regulators of biofilm formation *mrkA*, *wzm*, *wbbm*, and *treC*, the results showed that CA downregulated the expression level of *mrkA* and *wbbm* in five CRKP strains, suggesting that CA may regulate biofilm formation by regulating these factors. Besides, CA significantly inhibited the percent induction of luminescence of *V. harveyi*, but the AI-2 QS system network of *K. pneumoniae* is complex, the effect of CA on other factors of the QS system of *K. pneumoniae* type II and its mutual regulation with virulence factors need to be further explored in a future study. In view of the rapid increase of bacterial resistance, combining non-traditional antibiotic compounds and antibiotics as a new treatment regimen has thus garnered considerable scientific attention. We performed the checkerboard assays to evaluate the synergistic or additive effects between CA and known antimicrobials (meropenem, imipenem, ertapenem, ceftazidime, ciprofloxacin, levofloxacin, gentamicin, tobramycin, and ampicillin) on five CRKP strains in our study. The results showed that the CA/antimicrobial combinations exhibited a irrelevant effect on all five CRKP strains (data not shown). We believe that the development of molecular modifications in the future will help CA play a better role in clinical treatment, and we will pay more attention to this point in future research.

## 5 Conclusion

The present study found that CA can inhibit the virulence phenotype of CRKP and downregulate the expression level of the QS system and virulence-related genes. Because of the increase in the incidence of hv-CRKP in clinical settings, timely and accurate diagnosis of the infection and effective treatment are vital. As a new therapeutic drug for bacterial diseases, QSIs that can reduce the virulence of bacterial strains need to be further discussed in future research.

## Data availability statement

The original contributions presented in this study are included in the article/Supplementary material, further inquiries can be directed to the corresponding authors.

## Author contributions

LW conducted the experiments, analyzed the data, and wrote the manuscript. YZ participated in experiments. YL and MX took part in analysis of results. ZY and XZ participated in analysis of results. MS and TZ helped to design the study. All authors contributed to the article and approved the submitted version.

## Footnotes

1
http://blast.ncbi.nlm.nih.gov/Blast.cgi
