# Supplementary material for: Effects of chlorogenic acid on antimicrobial, antivirulence, and anti-quorum sensing of carbapenem-resistant Klebsiella pneumoniae
Source: Front Microbiol. 2022 Dec 13;13:997310. doi: 10.3389/fmicb.2022.997310 (PMC9793747; doi:10.3389/fmicb.2022.997310)
Supplement: Supplementary file 1 [file Table_1.docx]

**Table S2 Primer sequence, annealing temperature and production size used in this study**

| **Primer** | **Sequence** | **Annealing（℃）** |
| --- | --- | --- |
| *rmpA2* | F: 5‘- GTGCAATAAGGATGTTACATTA -3’  R: 5’- GGATGCCCCTCCTG -3’ | 53.3 |
| *iucA* | F: 5’- CTCAAGCTTAGTGCTTCCTGAATGCCT -3’ | 53.3 |
|  | R: 5’- CTCGGATCCTATGAGTCACCTGGTCAC -3’ |  |
| *luxs* | F: 5’- ACGCCATTACCGTTAAGATG -3’ | 55 |
|  | R: 5’- TGTCGTCAGCTCGTGTTATG -3’ |  |
| *mrkA* | F: 5’- AGCGATGCGAACGTTTACCTGTCTC -3’ | 60 |
|  | R: 5’- CGTCATCCTGTTTAGTGCCATCAGC -3’ |  |
| *wzm* | F: 5’- CTATCGAAGACGTATCCTTTAC -3’ | 58 |
|  | R: 5’- ATATTCTCACGCCCGGTAAG -3’ |  |
| *wbbM* | F: 5’- TTATCAGGCTGCCATTGCCAT -3’ | 56 |
|  | R: 5’- CAGCTATATGCCCAATAACGC -3’ |  |
| *treC* | F: 5’- ATGAGCCGCGATGTGTTTAC -3’ | 55 |
|  | R: 5’- TTTCAGCGCCACATAATCCG -3’ |  |
| *16srRNA* | F: 5’- CTGTGGATGCTCAAGGACTAC -3’ | 55.3 |
|  | R: 5’- ATCCCCACCTTCCTCCAGTT -3’ |  |
